# Strengthening Theoretical Testing in Criminology Using Agent-based Modeling

**DOI:** 10.1177/0022427814531490

**Published:** 2014-07

**Authors:** Shane D. Johnson, Elizabeth R. Groff

**Affiliations:** 1 UCL Department of Security and Crime Science, University College London, London, United Kingdom; 2 Department of Criminal Justice, Temple University, Philadelphia, PA, USA

**Keywords:** agent-based models, theory testing, empirical methods

## Abstract

**Objectives:**

*The Journal of Research in Crime and Delinquency* (*JRCD*) has published important contributions to both criminological theory and associated empirical tests. In this article, we consider some of the challenges associated with traditional approaches to social science research, and discuss a complementary approach that is gaining popularity—agent-based computational modeling—that may offer new opportunities to strengthen theories of crime and develop insights into phenomena of interest.

**Method:**

Two literature reviews are completed. The aim of the first is to identify those articles published in *JRCD* that have been the most influential and to classify the theoretical perspectives taken. The second is intended to identify those studies that have used an agent-based model (ABM) to examine criminological theories and to identify which theories have been explored.

**Results:**

Ecological theories of crime pattern formation have received the most attention from researchers using ABMs, but many other criminological theories are amenable to testing using such methods.

**Conclusion:**

Traditional methods of theory development and testing suffer from a number of potential issues that a more systematic use of ABMs—not without its own issues—may help to overcome. ABMs should become another method in the criminologists toolbox to aid theory testing and falsification.

## Introduction

Discussing the need for another journal dedicated to research in criminology, in the Foreword of the first issue of the *Journal of Research in Crime and Delinquency* (*JRCD*), the founding editor Lloyd E. Ohlin (1964:3) wrote “ … many practitioners will seize the opportunity to explore a journal where the frontier problems of theory and research in criminology are discussed.” Three of the most pressing contemporary problems in criminological enquiry are (1) the fuzziness of the theories, (2) the lack of data to test them, and (3) the nonlinear nature of the systems being modeled and the existence of feedback loops (both of which are difficult for statistical techniques to handle well—see Eck and Liu 2008). In this article prepared for the 50th anniversary issue of *JRCD*, we have two broad aims. The first is to classify the most influential contributions published in *JRCD* in the last 50 years and identify the theories that have so far received the most attention. The second is to examine the gaps in two of those theories that have thus far proven difficult test and consider how a research methodology at the frontier of the social sciences—agent-based computational modeling—might be employed to strengthen the specification of those theories and guide future data collection.

To provide a context for what follows, we begin with a brief discussion of methods commonly employed in criminological research, consider how agent-based modeling differs, and articulate how it can be used to test and strengthen criminological theories. We then present the results of two literature searches. The first considers the theoretical focus of the 50 most cited articles published in *JRCD* since its inception. The aim of doing so is to provide one perspective on which theories have received the most attention in *JRCD* publications over the last 50 years. The review is not intended to be exhaustive but rather to reflect the contribution of *JRCD* publications to the literature. In the next section, we examine which theories have received some form of testing using agent-based models (ABMs). To illustrate the potential value of ABMs, we then consider two theories that have received much attention in the criminological literature but little or none in modeling research. This approach allows us to elaborate upon the potential for ABMs to be used to explore theories that have been emphasized in past issues of *JRCD* but have not traditionally garnered attention from agent-based modelers.

## Traditional Social Science Research Methods

Over the last 50 years, researchers have used a variety of methods to test criminological theories. In broad terms, one can differentiate between qualitative and quantitative approaches, each of which has strengths and weaknesses. In this section, we will briefly mention those issues to frame the discussion that follows. We then consider what agent-based modeling is, how it compliments other approaches, and what it might contribute to theory testing, strengthening, and falsification.

Qualitative research encompasses a variety of methods (e.g., Bennett and Wright 1984; Cromwell 2006) that usually involve interactions (e.g., interviews, focus groups, etc.) with relatively small samples of participants. Such research has a particular strength in providing insight into how actors make decisions, and the process or mechanisms through which patterns of behaviors might emerge. It is an example of a so-called bottom-up approach in that the researcher is interested in testing or generating explanations as to why particular phenomena occur. Limited generalizability is a notable weakness, since probability samples cannot easily be drawn from the populations studied.

In contrast, quantitative methods take a top-down approach through the analysis of large samples of data (often collected using an explicit sampling frame) to look for regularities or patterns that are consistent with a particular theory or hypothesis. One of the most common quantitative modeling approaches employed in criminological enquiry uses some form of regression analysis. The idea behind these kinds of approaches is twofold. First, that the outcome or behavior of interest can be measured using an observable dependent variable, such as the volume of crime in an area. And, second, that an algebraic model can be constructed to represent the candidate theory of interest. The latter will comprise a set of explanatory variables—collected using some form of real-world sampling—considered to represent the key constructs of the theory in question. Hypothesis testing then involves estimation of the amount of variance in the dependent variable explained by the proposed model and the relative contribution of the independent variables. Considerable advances have been made in the maximum likelihood estimation methods employed in such modeling exercises (e.g., Osgood 2000; Rountree and Land 1996).

However, there are acknowledged problems with these two approaches to research, and hence many theories remain inadequately tested. One obvious limit is that correlation does not imply causality. A second is the lack of empirical data to accurately represent theoretical constructs of interest (see Sullivan and McGloin this issue). A third is that many theories involve complex dynamic interactions that evolve over time that cannot be *systematically* studied using the empirical methods described. In such cases, alternative methods are required.

In the next section, we describe how ABMs may help address some of the outstanding critical questions, allowing the exploration and strengthening of criminological theory. We are not suggesting such models should replace empirical investigations. Rather, that they may be used in combination, perhaps in an iterative fashion, to provide insight into phenomena of interest. For example, they offer an efficient way of ensuring that empirical investigations are well designed and that candidate theories are as well specified as possible. In the next few paragraphs, we describe ABMs to illustrate why they might be useful.

## ABMs as a Tool for Strengthening Criminological Theory

In their book on ABMs, Epstein and Axtell (1996) describe “a generative program for the social sciences and see the artificial society as its principle scientific instrument” (p. 177). This is a bold aspiration, but the principles that motivate it are those that most criminologists will agree with. The idea is simply this—if a theory is valid, then a formal implementation of it should be able to “grow” the outcomes the theory was developed to explain. This is a departure from statistical approaches for which something of a leap of faith is required to connect explanatory mechanisms to outcomes. The act of modeling a theory using an ABM requires concepts and mechanisms be formally articulated in a logical way. If this cannot be accomplished, doubt is cast on the veracity of the theory or its articulation (e.g., Benenson and Torrens 2004; Gilbert and Terna 1999; Gilbert and Troitzsch 2005). ABMs represent perhaps the most commonly used computational generative approach—and the simplest to understand—and hence, we concentrate on those here (for a collection of criminological examples, see Eck and Liu 2008).^1^

A typical ABM comprises three basic components—agents, rules, and an environment. Agents can represent anything of interest but are usually autonomous entities (e.g., offenders, citizens), whose behavior the researcher seeks to simulate. Over the many iterations of a simulation, agents engage in behaviors and interact with each other and their environment. Such behaviors are specified in a computer program that comprises among other things, *condition action rules* that guide agent decision-making. The program is intended to be a formal representation of the theory of interest (more on this below) and the rules used to specify agent behavior may reflect only a parsimonious representation of the theory concerned. Despite this, model outcomes can be complex and unexpected. One reason for this is that agents interact, and these interactions can affect subsequent choices and impact the environment. These interdependencies can generate feedback loops, for example, not explicitly included in the agent rule sets (but often observed in the real world). That is, complex behavior can *emerge* from simple rules. In terms of assessing simulation outcomes, the focus is not on the individual choices made by each agent (though these can be analyzed) but on the macro-level phenomena observed at the level of the overall system. Such outcomes would include documented regularities like the age–crime curve (Steffensmeier et al. 1989) or the finding that crime clusters in space (e.g., Braga, Papachristos, and Hureau 2010; Groff, Weisburd, and Yang 2010; Johnson and Bowers 2010; Weisburd, Bruinsma, and Bernasco 2009).

ABMs do not include a central controller that objectively assesses the situation each agent encounters. Instead, agent decision-making is autonomous and usually reflects a form of bounded rationality (Simon 1952). That is, agents generally make decisions based only on the information that is available to them and (in some models) past experience. Moreover, where an agent might select from two or more choices, the decision-making process will usually be stochastic so that a favored (or the most optimal) choice will not always be selected, just as in real life.

ABMs share some of the advantages of the empirical methods discussed previously but offer the promise of addressing some of their weaknesses. For example, in the spirit of qualitative research, they allow a researcher to formally specify theoretical mechanisms and see if they are *sufficient* to replicate findings observed in the real world (e.g., Epstein 2006). A researcher can also experiment by changing specific agent behaviors, or characteristics of the agents or their environment. As the researcher specifies the behaviors, threats to internal validity that are a common concern for empirical studies, such as spurious effects or unknown confounders, are minimized. Moreover, as with quantitative studies, simulations can be run for long simulated periods of time to generate large samples of data, but unlike empirical studies can easily (and should) be replicated many times.

A detailed account of how an ABM is constructed and tested is beyond the scope of this article, but a brief explanation will be helpful. The first stage is to formalize the theoretical model of interest (Gilbert and Terna 1999; Gilbert and Troitzsch 2005; Grimm and Railsback 2005) by translating it into a series of computer algorithms that represent key components. This can be difficult, as theories expressed in natural languages are often vague, ambiguous, and open to interpretation. But the process may be helpful regardless of whether an ABM is ultimately produced as the identification of elements of a theory that cannot be formalized can highlight aspects of it that require intellectual attention or data collection (Eck and Liu 2008). Considering this point, we wondered how many existing criminological theories would pass the formalization test. We do not have an answer to this question, but it is one worth asking.

ABMs can be produced in a number of ways, but freely available ABM platforms such as NetLogo (Wilensky 1999) or Repast (Collier 2003) are commonly used. Having implemented a formal model, model parameters require calibration. One appeal of ABMs is that heterogeneity can easily be incorporated. For example, reflective of actual people, agents may vary on particular attributes (e.g., age, self-control) and these may change over time. For instance, agents can learn, adapting their behavior to achieve particular goals, or some form of satisfaction (e.g., Axelrod 1997). Places too might vary in one or more ways, such as their social composition or signs of decay. Such factors can be dynamic, affected by agent activity or exogenous factors.

ABMs have limitations (Gilbert and Terna 1999; Gilbert and Troitzsch 2005). Similar to other types of modeling, findings are constrained by the assumptions and rules that underpin the model. An example is the calibration of an ABM. For instance, the values (or distribution of values) associated with particular parameters may be unknown. Choices made about how to represent those parameter values and the associated condition action rules will affect the process and the outcomes observed. In addition, the findings from an ABM must be interpreted conservatively since they do not represent an empirical test. Instead, they explore the “extent to which a theory is plausible” (Groff 2007:79; for a discussion of other issues of validity, see Berk 2008; Townsley and Johnson 2008).

On one hand, this can be problematic insofar as a model based on incomplete information may be of little value. On the other, ABMs can be tested using different values (or distributions) to identify the range of parameter values (or distributions) for which a particular model is able to *sufficiently* generate a particular outcome. Furthermore, where little is known about key model parameters, a research agenda can describe what data should be collected to inform (theory and) model development. Such an exercise can supplement research that uses methods other than simulation.

Qualitative/ethnographic research shares common ground with ABM in that both focus upon the mechanisms and processes through which outcomes emerge. This differs from quantitative research that typically looks for patterns that are consistent with a particular theory. Qualitative research offers an empirical complement to ABMs virtual societies, a relationship that can go both ways (Tubaro and Cassilli 2010). For example, qualitative research may provide insight into the decision-making of actors of interest, which can be incorporated into the condition action rules of an ABM. Such decision-making might already have been studied, or it might be identified as important through the process of formalizing an ABM. Further, an ABM can be used to examine how such condition action rules influence model outcomes, or how sensitive the model is to changes in the specific decision criteria (e.g., if particular tipping points need to be reached for an action to be triggered, see below), and so on. This can be a much more time and cost-effective strategy than undertaking additional interviews in a new population (Tubaro and Cassilli 2010). In this way, the approaches can (and should) be seen as reciprocal, and ABM can be considered another component of the researchers toolbox.

Put differently, ABM can increase the formality of qualitative research by making possible *in silica* experimentation—the agent’s “lives” can be restarted and allowed to play out under different assumptions. This might be used to test different theories or even interventions. For example, agents can provide estimates of the counterfactual by allowing outcomes to be modeled for those who do and do not receive “treatments.” The possibilities for qualitative research and ABM to strengthen one another are many. For a more in-depth treatment of this subject, see Tubaro and Cassilli (2010).

This same reciprocity applies to quantitative research. Consider Bruch and Mare’s (2006) investigation into Schelling’s (1971) classic model of ethnic segregation. Developed to explain the high levels of neighborhood segregation observed in American cities at the time, Schelling’s model explored whether uncoordinated^2^ activity on the part of residents could lead to unexpected aggregate behavior. Using a parsimonious spatial representation, Schelling showed that a simple “tipping point” model was able to reproduce highly segregated patterns of neighborhood segregation for a population of agents that were, at the individual level, happy to live in a mixed neighborhood. At the start of the simulation, agents—which represent households that belong to one of the two groups—are randomly placed on a regular grid that represents an abstract city. During each cycle of the simulation, every agent surveys its eight neighbors and computes what fraction belong to the same group. Where this fraction is lower than a given *tolerance* threshold, or tipping point, the agent moves to a new location. The model is run many times and each choice by every agent has the potential to affect those of the others by changing the composition of their neighborhoods; their behavior is interdependent. In experiments, Schelling and others used variants of the decision rules and showed that segregation can occur for tolerance thresholds that are substantially lower (i.e., as low as 30 percent) than the aggregate behavior of residents would otherwise suggest.

What is important for our discussion is that Schelling’s model has inspired the development of theoretical explanations and the collection of empirical data to test them. Bruch and Mare (2006) carefully elucidate the mechanisms underlying Schelling’s model and consider alternative implementations for which agent preferences are based on a continuous rather than discrete threshold function. Using an ABM, they show that different “tolerance functions” (e.g., a threshold function vs. a continuous function) generate different outcomes, even if the aggregate levels of tolerance computed across all agents are the same. This is useful in and of itself, but it also suggests an agenda for the collection and analysis of empirical data. In the same article, the authors analyze data collected for a large-scale survey. Results support the conclusions of their ABM.

This study illustrates the iterative nature of research and how ABMs can be used to help refine theory and to guide empirical research intended to test it. In this case, the ABM was used to test a hypothesis that would be time consuming to do empirically. Having established its plausibility, the researchers *then* examined the empirical record.

## Theoretical Focus of Articles in *JRCD*

We now consider the theoretical focus of the 50 most cited *JRCD* articles (1964–2012). Thirty-three of these tested one or more perspective using empirical data.^3^ Table 1 shows that the general theory of crime (Gottfredson and Hirschi 1990) was the most frequently tested, followed by general strain theory (Agnew 1992) and neighborhood/social disorganization theories (Sampson and Groves 1989; Shaw and McKay 1942). To estimate the relative popularity of the theories over time, we compute how many years after the date of the oldest article published (1979) each article appeared in *JRCD*, and take the average for the articles associated with each theory. Theories with averages closer to zero are those that were tested during the earliest period considered. On average, articles that tested the general theory of crime or neighborhood characteristics/social disorganization theories were published most recently, followed by those using routine activity/lifestyle/victimization theories.

**Table 1.** Theories Tested in *Journal of Research in Crime and Delinquency’s* (*JRCD*) Most Cited Articles.

Theory	Number	Average Age of Articles Relative to 1979
General theory of crime	8	18
General strain theory/strain	7	13
Neighborhood characteristics/social disorganization	6	18
Social control	4	14
Differential association	3	13
Fear of crime	3	7
Routine activity theory/lifestyle/victimization	3	16
Social learning	2	10
Other	13	11

## Theories Tested Using ABM

We next examined articles that used some type of simulation of urban crime. We conducted a systematic search of all articles written in the English language published (in any journal, book, thesis, etc.) through July 2012. A detailed list of search terms used and the databases searched is available from the authors upon request. A total of 36 publications met our search criteria. Table 2 shows that 33 of these mentioned some kind of theoretical framework. Of these, routine activity theory was modeled over twice as often as any other theory (*n* = 23). Crime pattern theory and the rational choice perspective were also frequently used. In contrast, social disorganization theory and social cohesion/collective efficacy were used (superficially) in only four models each. There was a definite bias toward opportunity theories and those appearing under the rubric of environmental criminology versus theories about individual human behavior (e.g., strain theory that appeared in only one article).

**Table 2.** Theoretical Framework Tested in Agent-based Models.

Theory	Number of Articles
Routine activity theory	23
Crime pattern theory	9
Rational choice perspective	8
Social disorganization	4
Social cohesion/collective efficacy	4
Beliefs/desires/intentions (BDI)	3
Near repeats	2
PECS	2
Situational crime prevention	2
Strain	1

*Note:* PECS = physical conditions, emotional states, cognitive capabilities, and social status.

This comparison shows that the criminological theories receiving more attention in the most highly cited articles in *JRCD* are precisely those that have received the least from the computational modeling community. Why have those involved in computational modeling embraced theories of environmental criminology? One reason is that simulation models typically model dynamic events, and do so in an iterative fashion. Theories of environmental criminology, such as routine activity (Cohen and Felson 1979) and crime pattern theory (Brantingham and Brantingham 1984) focus on crime at the event level, and consider the necessary ecological conditions for a crime to occur at a particular place and time. Consequently, they are expressed in a form that is directly compatible with the ABM approach (for further discussion, see Brantingham and Brantingham 2004).

We now turn to two examples of theories, prominent in *JRCD* articles that have received little attention from computational modelers but might benefit from it.

## General Strain Theory—What Can ABM Contribute?

In contrast to theories of the crime event, general strain theory (GST) considers how individual differences affect offender motivation. In particular, Agnew (1999:372) suggests that “strain may involve the removal of positively valued stimuli.…and negative relations with parents, teachers, peers and others -with such relations involving insults, verbal threats, and other noxious behavior. These types of behavior increase the likelihood that the individual will experience negative affect.… This creates pressure for corrective action, and delinquency is one response” (Agnew, 1995, p. 372). Thus, GST considers how an offender might become motivated to commit a crime but not when and where they might do so. The theory is largely silent on crime events.

The description quoted previously clearly outlines a dynamic process whereby a person’s interaction with others influences the strain they experience. While data could be (and are) collected using surveys to estimate how such experiences shape an offender’s propensity to offend, it is difficult to see how sufficiently complete data could be collected to allow an adequate test of the theory. On the other hand, in an ABM, such incidents could be recorded and their impact on simulation outcomes observed.

In translating the theory into an ABM, it would be possible to specify rules about how agents interact with each other and their environment and how their interactions provide triggers that influence the amount of strain experienced. Such a model would need to articulate each of the different sources of strain. It would also need to specify how much strain experienced would likely to lead to delinquency, whether a threshold or some other function would apply (see above), how this might vary across agents, and what factors might mediate it. Some of these issues have been discussed in the literature (e.g., Agnew 2001, 2012), but perhaps not so comprehensively that they could be formally expressed at the level of specificity necessary for an ABM. For example, are some sources of strain more damaging than others or more damaging to some people than others? At what rate do different sources of strain build up? Are cumulative effects linear? Are there tipping points? Do the effects of some (or all) sources of strain decay with time? Do some (or all) sources of strain have long-term effects, and do some have abrupt but temporary effects?

Another element of GST concerns the way in which a person’s relations to their parents and others might mediate how strain experienced influences behavior. To model these effects, researchers would need to be specific about how such influences operate. They might also want to specify how people’s social networks impact upon them more broadly.

Formally articulating these aspects of the theory would represent a useful step in developing an ABM and in strengthening the theory. Given the number of influences considered in GST, modeling it would be a complicated task. However, ABM architectures such as physical conditions, emotional states, cognitive capabilities, and social status (PECS) may provide a convenient framework to support the modeling of such theories (e.g., Urban 2000).

## Social Disorganization Theory—What Can ABM Contribute?

Sampson and Groves (1989:777) explain that “[i]n general terms, social disorganization refers to the inability of a community structure to realize the common values of its residents and maintain effective social controls.” Moreover, “the structural dimensions of community social disorganization can be measured in terms of the prevalence and the interdependence of social networks in a community—both informal (e.g., friendship ties) and formal (e.g., organizational participation)—and in the span of collective supervision that the community directs toward local problems.” Emergence of the cohesion necessary to support collective action is affected by a community’s socioeconomic characteristics and its residential stability.

Although Sampson and colleagues have specified the mechanisms from which collective action emerges, accurate measurement of those mechanisms remains elusive. Researchers have attempted to measure community social organization through surveys of individuals and scholars have created an entire methodology, systematic social observation, in an effort to quantify key elements (Sampson and Raudenbush 1999). This has revealed some very interesting patterns in one city, Chicago, IL. Unfortunately, the method is costly and even with improved technology is likely beyond the reach of cash-strapped funding agencies.

ABM can help to test social disorganization theory by representing the individual-level dynamic processes of friendship tie formation, participation in neighborhood organizations, and how these enable a *community* to maintain effective social control and reflect the goals of its residents. To capture the dynamics, such a model might represent both individuals and neighborhoods as agents with characteristics that change over time. Agent decisions could be influenced by other individuals with whom they interact and the neighborhood in which they occur. In addition, individual agents could “observe” decisions their neighbors make, which could inform their personal perception of the neighborhood and their subsequent decisions. With repeated encounters, agents could begin to recognize each other. Over time, they might begin to speak and get to know one another. The strength of an agent’s social ties could increase, as they become familiar with more of their neighbors. Development and changes in social ties could be monitored in real time.

But many details of the theory remain unspecified. At what point does someone begin to understand neighborhood social norms? Must they observe active instances of intervention or is the absence of behavior evidence of what is (not) acceptable in that neighborhood? What is the number and/or the strength of social ties that are necessary before a neighborhood becomes socially organized? How many individuals need to participate in community organizations? What is the interaction between the strength or number of social ties among neighbors and participation in community organizations that enables social organization to emerge? ABM offers a robust platform for thinking through the answers to these questions, operationalizing them within an artificial world, and examining the sensitivity of the model to changes in associated parameters.

## Conclusion

In this article, we have discussed two traditional approaches to social science research and highlighted how ABM offers complementary strengths to those wishing to test and strengthen criminological theory. Our review of the most cited articles published in *JRCD* in the last 50 years revealed the field’s interest in dispositional theories of crime, although ecological theories have received more attention recently. At the same time, agent-based modelers have focused on the criminal event and relied on Cohen and Felson’s (1979) routine activity theory, the Brantinghams’ crime pattern theory, and Clarke and Cornish’s rational choice perspective. Here, we argue that agent-based computational modeling could play a wider role in testing criminological theory if it is applied to a greater variety of theoretical perspectives.

Our brief discussion of how GST and social disorganization theory might benefit from ABMs is illustrative. We do not suggest these are the only theories that could or should be examined. We believe we have only scratched the surface. As *JRCD* moves forward into the next 50 years, consideration of new methods for strengthening criminological theory is critical to Lloyd E. Ohlin’s (1964:3) vision of a journal “where the frontier problems of theory and research in criminology are discussed.”

## Notes

1. Traditional simulation encapsulates a broad field that includes statistical modeling techniques such as Monte Carlo methods that employ resampling and permutation approaches (for an example of such methods in criminology, see Johnson 2009). Simulation also includes a variety of models such as dynamic systems, queuing models, and microsimulation that focus on process. For a good introduction to these methods, see Gilbert and Troitzsch (2005). ABMs differ in that they allow for heterogeneity and for dynamic individual decision making that can be influenced by the situation and agent interaction.

2. Uncoordinated in that no central controller—or invisible hand—directs agent actions.

3.
*Journal of Research in Crime and Delinquency*’s (*JRCD*) managing editor provided a list of the top 50 most cited articles in April 2013. The abstracts of all articles were reviewed and categorized by types of theories tested. The list of articles is available from the authors.
